# Diacylglycerol Acyltransferase-1 (DGAT1) Inhibition Perturbs Postprandial Gut Hormone Release

**DOI:** 10.1371/journal.pone.0054480

**Published:** 2013-01-15

**Authors:** Hua V. Lin, Dunlu Chen, Zhu Shen, Lei Zhu, Xuesong Ouyang, Aurawan Vongs, Yanqing Kan, John M. Levorse, Edward J. Kowalik, Daphne M. Szeto, Xiaorui Yao, Jianying Xiao, Shirley Chen, Jinqi Liu, Marga Garcia-Calvo, Myung K. Shin, Shirly Pinto

**Affiliations:** 1 Merck Research Laboratories, Rahway, New Jersey, United States of America; 2 Kenilworth, New Jersey, United States of America; Tohoku University, Japan

## Abstract

Diacylglycerol acyltransferase-1 (DGAT1) is a potential therapeutic target for treatment of obesity and related metabolic diseases. However, the degree of DGAT1 inhibition required for metabolic benefits is unclear. Here we show that partial DGAT1 deficiency in mice suppressed postprandial triglyceridemia, led to elevations in glucagon-like peptide-1 (GLP-1) and peptide YY (PYY) only following meals with very high lipid content, and did not protect from diet-induced obesity. Maximal DGAT1 inhibition led to enhanced GLP-1 and PYY secretion following meals with physiologically relevant lipid content. Finally, combination of DGAT1 inhibition with dipeptidyl-peptidase-4 (DPP-4) inhibition led to further enhancements in active GLP-1 in mice and dogs. The current study suggests that targeting DGAT1 to enhance postprandial gut hormone secretion requires maximal inhibition, and suggests combination with DPP-4i as a potential strategy to develop DGAT1 inhibitors for treatment of metabolic diseases.

## Introduction

Obesity, characterized by excessive fat accumulation in the form of triglycerides (TG) in adipose tissue and ectopic TG deposition in other organs, is a primary risk factor for type 2 diabetes, cardiovascular diseases, and other comorbidities. Current pharmacological treatments are inadequate for weight loss [1]. Thus, a more detailed knowledge of pathways that influence energy homeostasis is necessary for the development of safe, more effective obesity and diabetes therapies.

The final step of TG biosynthesis, the joining of diacylglycerol with fatty acyl CoA, is catalyzed by diacylglycerol O-acyltransferase (DGAT) enzymes. Of the two DGATs identified to date, DGAT1 garnered much attention as a target for the treatment of obesity since DGAT1 knockout mice showed marked resistance to diet-induced obesity [2]. However, the precise mechanisms responsible for the lean phenotype in DGAT1 knockout mice remain elusive, in large part due to a host of abnormalities associated with complete DGAT1 deficiency, including increased locomotor activity [2], a severe defect in skin lipid metabolism [3], and increased thermogenesis and dissipation of body heat [4]. DGAT1 is broadly expressed, with the highest level of expression in intestinal epithelium, adipose tissue, and liver [5]. DGAT1 activity in the intestine is thought to play a key role in regulating systemic metabolism based on the observation that DGAT1 knockouts showed markedly reduced plasma TG levels following an oral lipid challenge [6]. In addition, intestine-only DGAT1 reconstitution in DGAT1 knockout mice restored their sensitivity to diet-induced obesity [7]. Therefore, inhibiting DGAT1 specifically in the intestine may have the potential to reduce body weight. DGAT1 in other tissues also play important roles in regulating energy homeostasis. Transplantation of DGAT1-deficient white adipose tissue (WAT) into wild type mice reduced adiposity [8]. Conversely, DGAT1 overexpression in skeletal muscle [9] or in macrophages [10] improved insulin sensitivity. Thus, the net metabolic consequence of global DGAT1 inhibition is unclear. Furthermore, the complex phenotypes of DGAT1 knockout mice raise safety concerns for the development of pharmacological agents that lead to maximal, global DGAT1 inhibition. Therefore, gut-specific and/or partial DGAT1 inhibition may be attractive approaches to circumvent potential adverse effects associated with global DGAT1 inhibition. Several small molecule DGAT1 inhibitors have been reported to ameliorate obesity in rodents [11]–[15]. However, the contributions of gut vs. systemic DGAT1 inhibition as well as the degree of DGAT1 inhibition required for these effects have not been elucidated.

It was recently shown that oral lipid-induced elevation in the incretin glucagon-like peptide-1 (GLP-1) was prolonged in DGAT1 knockout mice [16] and in rodents treated with DGAT1 inhibitors [17], [18](Liu et al., submitted). Therapies targeting the GLP-1 pathway, including GLP-1 receptor agonists and inhibitors of the GLP-1 degrading enzyme dipeptidyl-peptidase-4 (DPP-4), reduce glycemia in diabetic patients [19], [20]. In addition, GLP-1 receptor agonists, but not DPP-4 inhibitors lead to sustained weight loss. Therefore, understanding the effect of DGAT1 inhibition on the GLP-1 pathway will help elucidate its therapeutic potential in the clinic.

In this study, we provide genetic and pharmacological evidence for a key role of intestinal DGAT1 in regulating postprandial triglyceridemia and gut hormones following ingestion of lipid-rich meals. Our data indicate that maximal inhibition of DGAT1 may be required for efficacy and suggest a potential strategy of combining with DPP-4 inhibitors for the treatment of obesity and diabetes.

## Results

### Gene dosage effects on DGAT1 expression and functional activity

**Figure 1:**
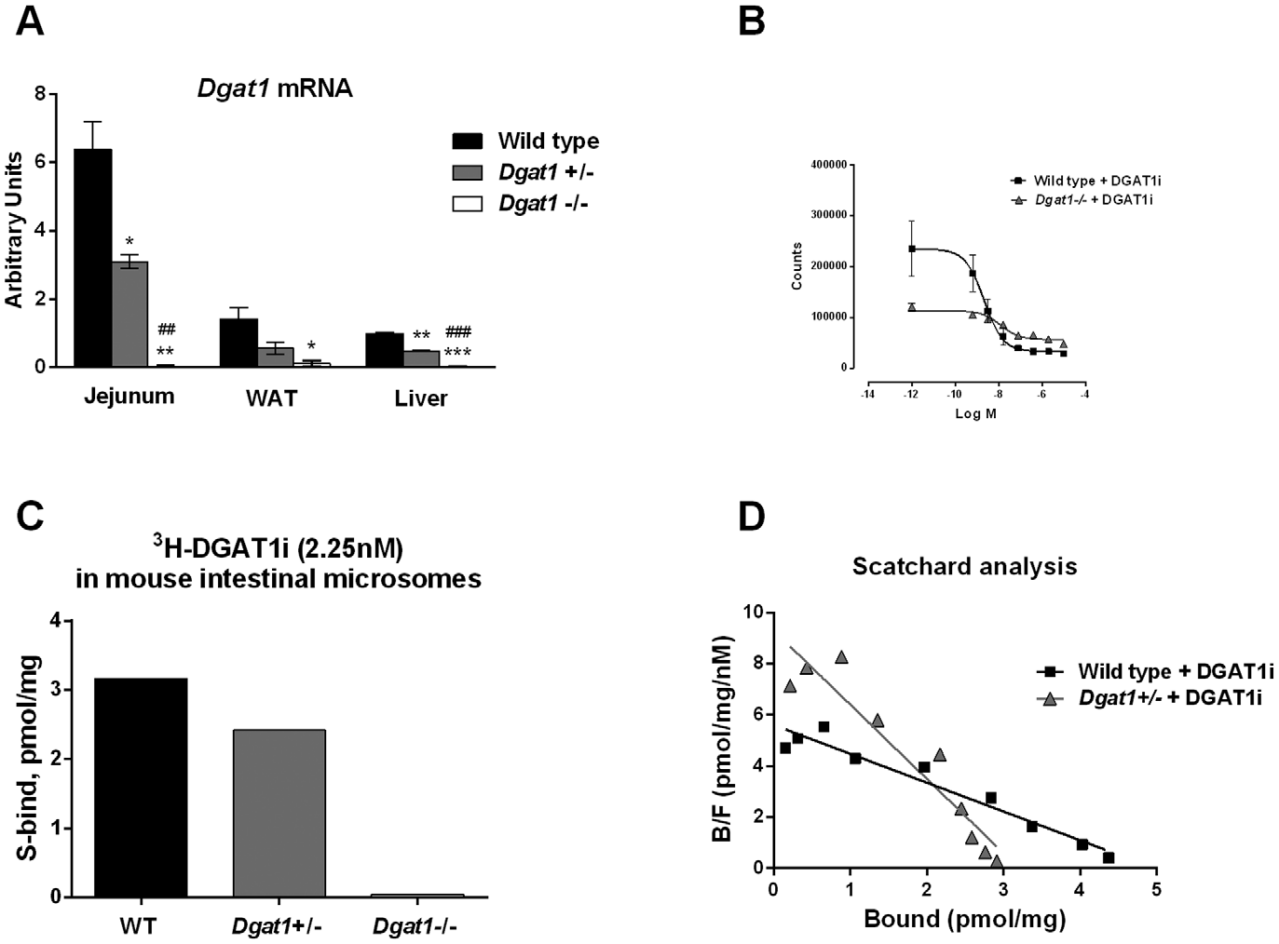
Biochemical characterization of DGAT1 heterozygous and homozygous knockout mice. (A) DGAT1 mRNA expression determined by quantitative RT-PCR in jejunal mucosa, white adipose tissue, and liver from *Dgat1*+/−, *Dgat1*−/−, and age-matched C57BL/6N wild type mice. N = 3. DGAT1 mRNA and were normalized against β-actin mRNA. All data are mean ± SEM. *P<0.05, **P<0.01, ***P<0.001 vs. wild type. ^##^P<0.01, ^###^P<0.001 *Dgat1*−/− vs. *Dgat1*+/−. (B) DGAT enzymatic activity in intestinal mucosa of *Dgat1*−/− and wild type mice measured by TG synthesis in the presence of various concentrations of DGAT1i. (C) Binding of intestinal microsomes of wild type, *Dgat1*+/−, and *Dgat1*−/− mice to ^3^H-labeled DGAT1i. Microsome preps pooled from 2–5 mice were used in the assay. (D) Scatchard analysis of ^3^H-labeled DGAT1i binding in intestinal microsomes of wild type and *Dgat1*+/− mice.

To understand the degree of DGAT1 inhibition required for improving metabolic parameters, we used a genetic approach to determine the metabolic impact of complete or partial DGAT1 loss-of-function. We first examined mice lacking one or both copies of *Dgat1* (*Dgat1*+/− and *Dgat1*−/−). Body weights of *Dgat1*+/− and *Dgat1*−/−mice were not significantly different from that of wild type controls when fed a normal chow diet [2] (data not shown). As expected, DGAT1 mRNA expression in small intestine, WAT, and liver showed 51–62% reductions in *Dgat1*+/− mice compared to wild type controls, and was undetectable in *Dgat1*−/− mice (Figure 1A). To determine whether the amount of DGAT1 mRNA correlates with the level of its functional activity, we examined DGAT enzymatic activity in intestinal mucosa, where DGAT1 showed the highest enrichment at the mRNA level. Using reaction buffers containing high magnesium chloride that would inhibit DGAT2 activity and a thin layer chromatography-based approach, DGAT activity in intestinal mucosa of wild type mice showed dose-dependent inhibition by a DGAT1-specific inhibitor (DGAT1i) [21], with an IC_50_ of 2.2 nM and maximal inhibition of 88% (Figure 1B). DGAT activity in intestinal mucosa harvested from *Dgat1*−/− mice was significantly reduced compared to wild type controls but not absent. These data are consistent with DGAT1 acting as the predominant DGAT enzyme in the ex vivo enzymatic assay performed. However, we cannot exclude a potential contribution of DGAT2 or other enzymes to intestinal DGAT activity. The residual TG biosynthetic activity observed in *Dgat1*−/− mice and in wild type mice following addition of DGAT1i imply additional enzymes with DGAT-like activities may contribute to TG synthesis in the small intestine. To further characterize expression level of DGAT1 as it relates to the genotype, ^3^H-labeled DGAT1i was titrated into intestinal mucosa extracted from wild type, *Dgat1*+/−, and *Dgat1*−/−mice. *Dgat1*−/− tissue showed a complete lack of binding to DGAT1i, while *Dgat1*+/− tissue binding appeared to be reduced relative to wild type (Figure 1C). To quantify DGAT1i binding sites in the intestinal mucosa, a Scatchard analysis was performed (Figure 1D), and Bmax was calculated to be 4.8 pmol/mg and 2.9 pmol/g for wild type and *Dgat1*+/− tissue, respectively. These data indicate that DGAT1 mRNA expression correlates well with DGAT1 enzyme levels in intestinal mucosa and are consistent with a partial reduction of DGAT1 enzyme in *Dgat1*+/− mice.

### DGAT1 gene dosage effects on postprandial triglyceridemia, gut hormones, and gastric emptying

**Figure 2:**
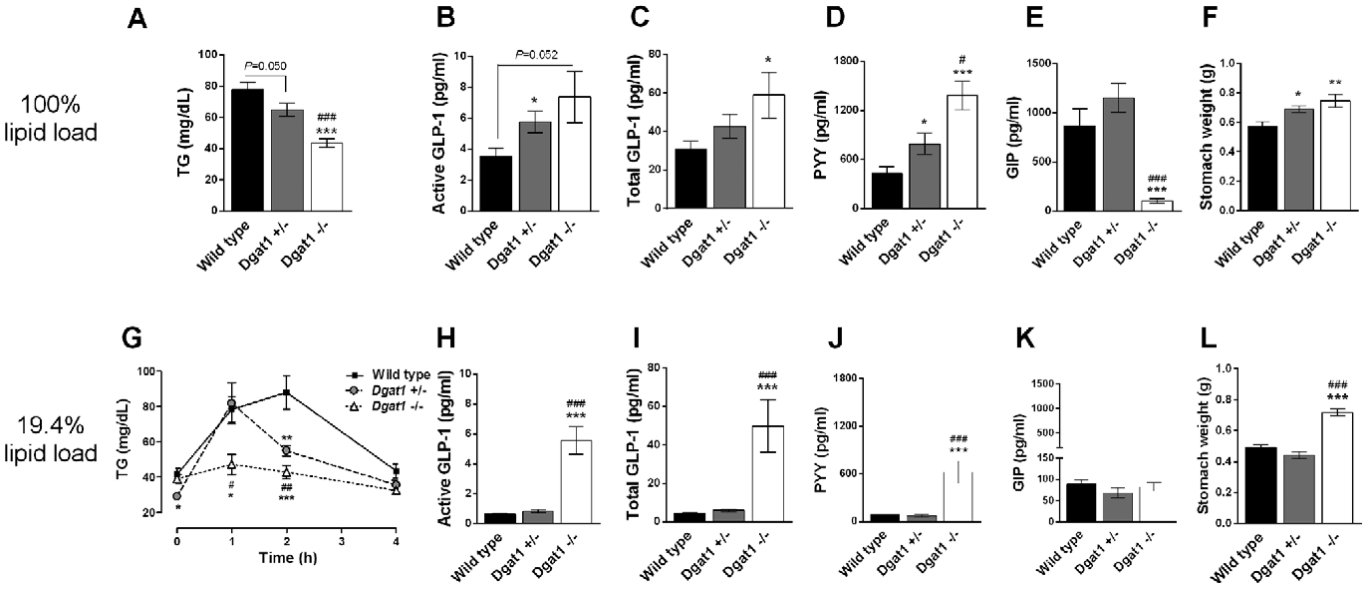
Postprandial triglyceridemia, gut hormones, and gastric emptying in *Dgat1*+/− and *Dgat1*−/− mice. 4- to 5-month-old *Dgat1*+/−, *Dgat1*−/−, and wild type mice maintained on normal chow diet were fasted overnight, then p.o. dosed with (A–F) corn oil (100% lipid load, 8.14 kcal/ml) or (G–L) a mixed meal containing corn oil:Ensure-plus = 3∶17 v/v (19.4% lipid load, 2.48 kcal/ml) at 10 ml/kg body weight. At 2 h after meal challenge, plasma levels of (A) TG, (B, H) active GLP-1, (C, I) total GLP-1, (D, J) PYY, (E, K) GIP, and (F, L) stomach weight were measured. (G) In a separate cohort, plasma TG at 0, 1, 2, and 4h after 19.4% lipid load was determined. N = 8–12. *P<0.05, **P<0.01, ***P<0.001 vs. wild type. ^#^P<0.05, ^##^P<0.01, ^###^P<0.001 *Dgat1*−/− vs. *Dgat1*+/−.

*Dgat1*−/− mice showed markedly reduced plasma TG and prolonged elevations in plasma GLP-1 and PYY, attenuated glucose-dependent insulinotropic peptide (GIP) induction, and delayed gastric emptying following an oral lipid challenge [16]. We examined the gene dosage effects of DGAT1 on these parameters. *Dgat1*+/−, *Dgat1*−/−, and wild type mice were given an oral bolus of 100% corn oil, henceforth referred to as 100% lipid load. Plasma TG at 2 h after oil challenge tended to be suppressed in *Dgat1*+/− compared to wild type controls, and was significantly lower in *Dgat1*−/− mice compared to both *Dgat1*+/− and wild type mice (Figure 2A). Plasma GLP-1 and PYY at 2h after oil challenge showed gene dosage-dependent increases in *Dgat1*+/− and *Dgat1*−/− mice compared to controls (Figure 2B–D). Gastric emptying, as measured by stomach weight, was delayed to a similar extent in *Dgat1*+/− and *Dgat1*−/− mice compared to controls (Figure 2F). Interestingly, oil-induced GIP was significantly attenuated in *Dgat1*−/− mice, but not affected in *Dgat1*+/− mice (Figure 2E). These data indicate that partial reduction in DGAT1 activity is sufficient to suppress plasma TG and lead to prolonged release of GLP-1 and PYY following an oil challenge, but is insufficient to suppress GIP. It was unclear whether DGAT1 also plays a role in modulating plasma TG and gut hormones after ingestion of a meal with lower lipid load that is more physiologically relevant. To address this question, we performed meal tolerance tests using an admixture of corn oil and Ensure-plus (3:17 v/v) that contains 19.4% of the lipids present in equal volume of pure oil. This resulting mixed meal contains 64%kcal from fat and 24.8 kcal/kg BW, which has similar %kcal from fat and total calorie content to a typical meal (200–250 mg) consumed by mice on high-fat diet (HFD) [22], [23] (60%kcal from fat and ∼26 kcal/kg BW). Thus, we consider the 19.4% lipid load to be at the high end of a physiologically relevant lipid-rich meal (see Table 1 for comparison of all dietary treatments used). Similar to what was observed with 100% oil, *Dgat1*+/− mice showed an intermediate suppression of plasma TG after meal (Figure 2G). Interestingly, following 19.4% lipid load, while *Dgat1*−/− mice showed significantly elevated GLP-1 and PYY and delayed gastric emptying (Figure 2H–J, L), none of these parameters were affected in *Dgat1*+/− mice compared to wild type controls. Of note, plasma GIP levels in wild type mice following 19.4% lipid load were 90% lower than those after 100% oil (Figure 2E, K), and were not further suppressed in *Dgat1*+/− or *Dgat1*−/− mice. Taken together, these data suggest that after a physiological meal, the residual DGAT1 activity in *Dgat1*+/− mice is above the threshold to maintain normal postprandial GLP-1, PYY, and gastric emptying. Conversely, 100% oil challenge presents substantially more substrates (fatty acids and monoacylglycerols) in the intestine and thus requires higher DGAT1 capacity for effective TG resynthesis and appropriate regulation of gut hormones and gastric emptying.

**Table 1:** Energy content analysis of dietary treatments.

Dietary treatment	Ingredients (vol:vol)	kcal in meal challenge per kg BW	kcal as lipids per kg BW	kcal as proteins per kg BW	kcal as carbohydrates per kg BW	Lipid amount (% isovolumic CO
2.6% lipid	1:1 H2O:EP	7.4	2.1	1.1	4.2	2.6
5.2% lipid	pure EP	14.8	4.2	2.2	8.4	5.2
19.4% lipid	3:17 CO:EP	24.8	15.8	1.9	7.2	19.4
28.9% lipid	1:3 CO:EP	31.4	23.5	1.7	6.3	28.9
52.6% lipid	1:1 CO:EP	48.1	42.8	1.1	4.2	52.6
68.4% lipid	2:1 CO:EP	59.2	55.7	0.7	2.8	68.4
100% lipid	pure CO	81.4	81.4	0	0	100
High-fat diet	RD D12492	26.2 (0.25g meal in mouse with 50g BW)	15.7	5.2	5.2	N/A

CO: corn oil, 8.14 kcal/ml, 100%kcal from fat. EP: Ensure-plus, 1.48 kcal/ml, 28.5%kcal from fat.

### Lipid-dependent effects of DGAT1 inhibition on postprandial GLP-1

**Figure 3:**
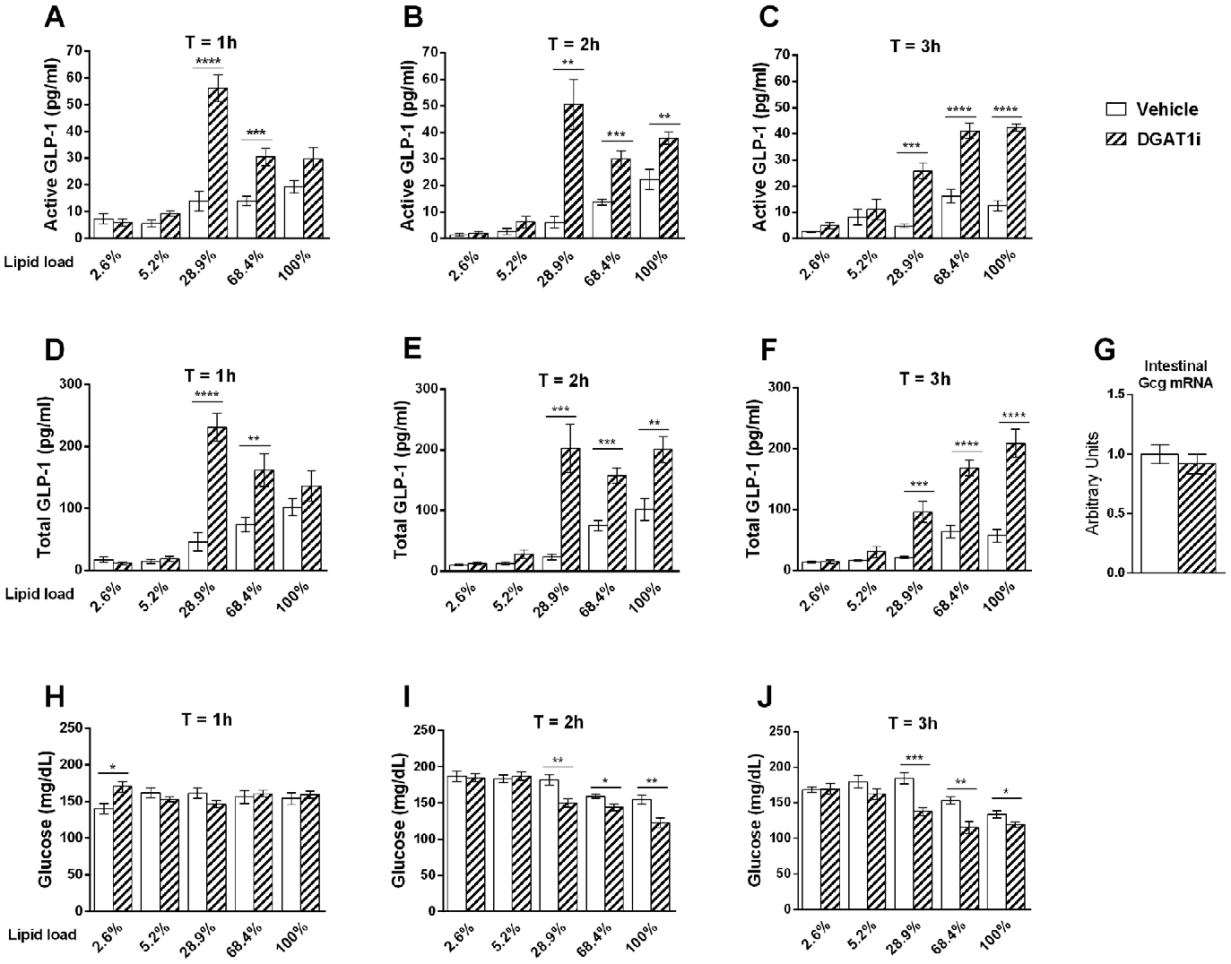
Effects of DGAT1 inhibition on postprandial GLP-1. 3-month-old lean C57BL/6N mice were p.o. dosed with DGAT1i at 3 mg/kg body weight and fasted overnight. The next morning, mice were p.o. dosed with mixed meals containing 2.6 (water:Ensure-plus = 1:1), 5.2, 28.9, 68.4, or 100% (corn oil:Ensure-plus = 0∶1, 1∶3, 2∶1, 1∶0 v/v) lipid load at 10 ml/kg body weight (0.74, 1.48, 3.15, 5.92, or 8.14 kcal/ml). At 1, 2, or 3h after meal challenge, plasma levels of (A–C) active GLP-1, (D–F) total GLP-1, and (H–J) blood glucose were measured. (G) 3-month-old lean C57BL/6N mice were p.o. dosed with 3 mg/kg DGAT1i or vehicle and fasted overnight. 18h after dosing, jejunal mucosa was harvested, and Gcg mRNA expression was measured by realtime PCR. Data are normalized against β-actin mRNA. N = 8. *P<0.05, **P<0.01, ***P<0.001, ****P<0.0001 between groups.

To better understand the interaction between meal lipid content and DGAT1 activity in postprandial GLP-1 regulation, we used DGAT1i to inhibit DGAT1 activity pharmacologically in wild type mice and performed lipid titration studies. Pilot studies showed that 3 mg/kg DGAT1i pretreatment at -18 h in mice completely suppressed TG excursion after oil challenge (data not shown), consistent with near-complete DGAT1 inhibition. Mice pretreated with vehicle or DGAT1i were orally dosed a mixed meal containing increasing lipid load ranging from 2.6 to 100%, and plasma levels of active and total GLP-1 were measured at various time points after meal challenge. DGAT1i had no effect on meal-induced GLP-1 in the presence of 2.6 or 5.2% lipid load, but led to significant elevations in GLP-1 when meal lipid load exceeded 19.4% (Figure 3A–C and data not shown) at 1, 2, and 3 h after meal challenge. The effect of DGAT1i on total GLP-1, which includes both the active forms and the inactive forms resulting from DPP-4 cleavage, mirrors that on active GLP-1 (Figure 3D–F). Furthermore, DGAT1i pretreatment at -18 h did not affect mRNA expression of Preproglucagon (Gcg), which encodes GLP-1 in intestinal L cells, in intestinal mucosa (Figure 3G). Taken together, these data provide further evidence that DGAT1 inhibition stimulates and prolongs postprandial GLP-1 secretion only when meal lipid content exceeds a certain threshold, without affecting Gcg transcription or the stability of active GLP-1 after secretion. Consistent with the incretin effect of GLP-1, DGAT1i-treated mice receiving a meal with lipid load of 28.9% or above showed significant reductions in plasma glucose at 2 and 3h (Figure 3H–J).

### Effects of gut-selective DGAT1 deficiency on postprandial triglyceridemia and gut hormones

**Figure 4:**
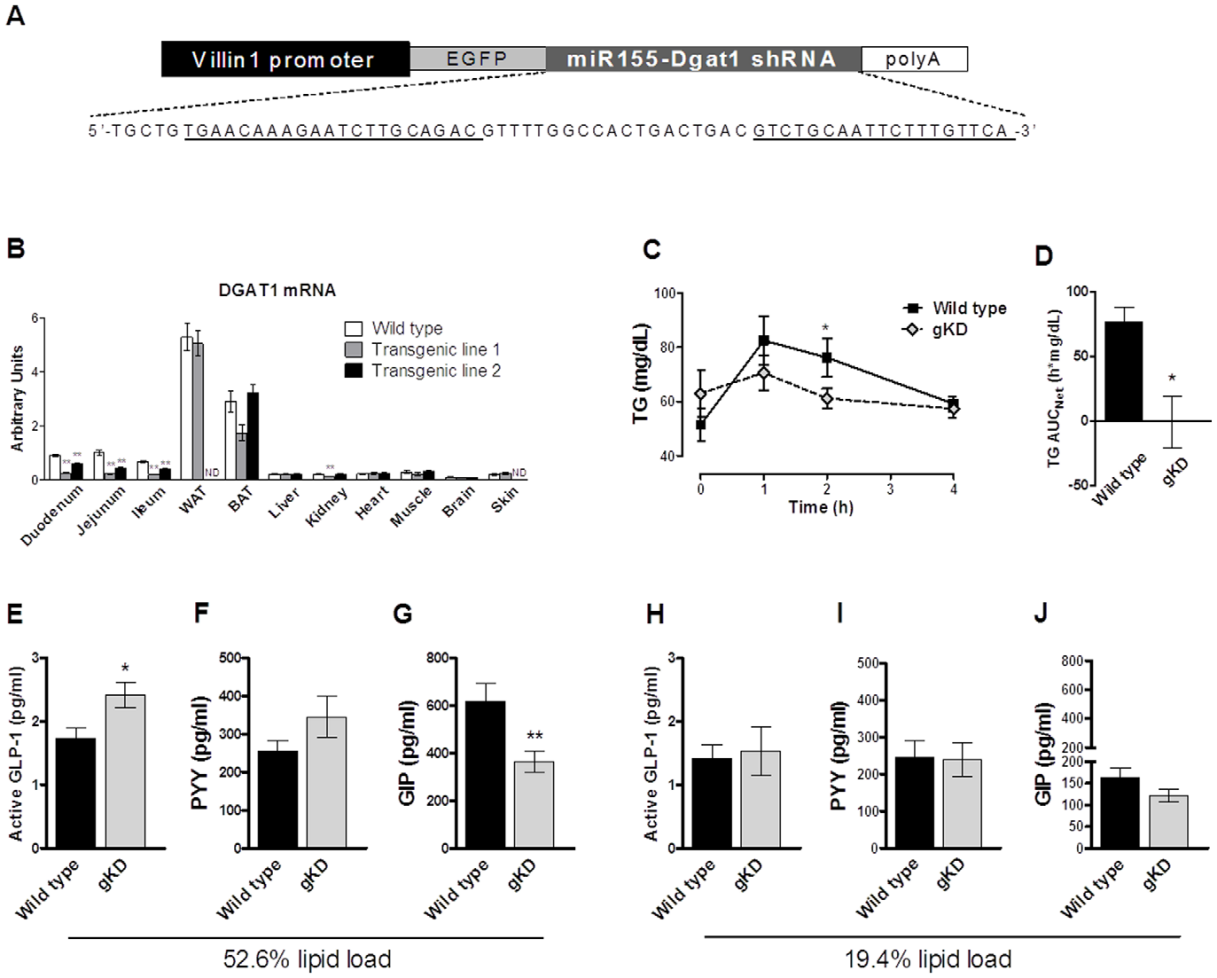
Gut-selective DGAT1 knockdown and its effects on TG and gut hormones. (A) Design of the transgenic construct used to generate DGAT1 gKD mice. A transgenic vector was constructed that contains a 12.4 kb *Villin1* promoter, a sequence encoding EGFP, followed by miR155-embedded *Dgat1* shRNA and a poly-A tail. Sequence of the miR155-embedded *Dgat1* shRNA is shown, with the sequences homologous to *Dgat1* underlined. (B) DGAT1 mRNA expression in selected tissues from two DGAT1-shRNA transgenic lines and wild type control littermates. N = 3–5. Data are normalized against Rplp0 mRNA. ND: not determined. (C, D) 4-month-old female gKD mice and wild type littermates were fasted overnight, then p.o. dosed with corn oil at 10 ml/kg. Plasma TG was measured at 0, 1, 2, and 4 h after oil challenge, and net area-under-the-curve (AUC) compared to time 0 was calculated. Overnight fasted gKD mice and wild type littermates were p.o. dosed with a mixed meal containing (E–G) corn oil:Ensure-plus = 1∶1 (52.6% lipid load, 4.81 kcal/ml) or (H–J) corn oil:Ensure-plus = 3∶17 (19.4% lipid load) at 10 ml/kg. Plasma levels of active GLP-1, PYY, and GIP were measured at 2 h after meal challenge. N = 10–14. *P<0.05, **P<0.01 vs. wild type.

Gut-specific reconstitution of DGAT1 restored postprandial TG excursion and the susceptibility to diet-induced obesity in *Dgat1*−/− mice [7], suggesting gut DGAT1 deficiency is *necessary* for the TG and body weight phenotypes in DGAT1 knockouts. However, postprandial gut hormone regulation was not assessed in this model. Furthermore, it was unclear whether gut DGAT1 deficiency is *sufficient* to confer metabolic benefits, and by inference, whether gut-selective DGAT1 inhibitors would be efficacious in treating metabolic disorders. To address these questions, we generated a transgenic mouse model with gut-selective DGAT1 knockdown. The transgenic construct contains the 12.4 kb *Villin1* promoter/enhancer that was previously shown to drive expression in intestinal epithelium [24], upstream of EGFP and a sequence encoding a *Dgat1*-targeting miR155-embedded shRNA (Figure 4A). Of the resulting founders, one line displayed ∼75% reductions in DGAT1 mRNA in intestinal mucosa, while a second line showed 35–54% reductions (Figure 4B). DGAT1 expression was not affected in WAT, brown adipose tissue (BAT), liver, skeletal muscle, heart, brain, or skin of transgenic mice compared to wild type littermates (Figure 4B). Consistent with other transgenic lines generated with *Villin1* promoter with sporadic expression in kidney [7], [25], DGAT1 mRNA expression in kidney was reduced by 48% in the first, but not the second transgenic line (Figure 4B). The first line with more effective DGAT1 knockdown in the intestine was chosen for all further analyses and is referred to as gut-selective knockdown (gKD) mice henceforth, although we cannot formally rule out potential effects of renal DGAT1 deficiency.

Plasma TG excursion following a 100% lipid load was significantly suppressed in gKD mice (Figure 4C, D). 2 h after a meal challenge containing 52.6% lipid load, gKD mice showed significantly increased active GLP-1, a trend toward increased PYY, and a significant attenuation of GIP induction compared to wild type littermates (Figure 4E–G), while these effects were not observed following 19.4% lipid load (Figure 4H–J). These data indicate that ∼75% gut-selective DGAT1 deficiency can increase postprandial GLP-1 only in the presence of supraphysiological lipid content. In addition, the difference in the GIP responses in *Dgat1*+/− (Figure 2E) and gKD mice (Figure 4G) suggests postprandial GIP regulation is sensitive to DGAT1 deficiency at a threshold between 50% and 75%.

**Figure 5:**
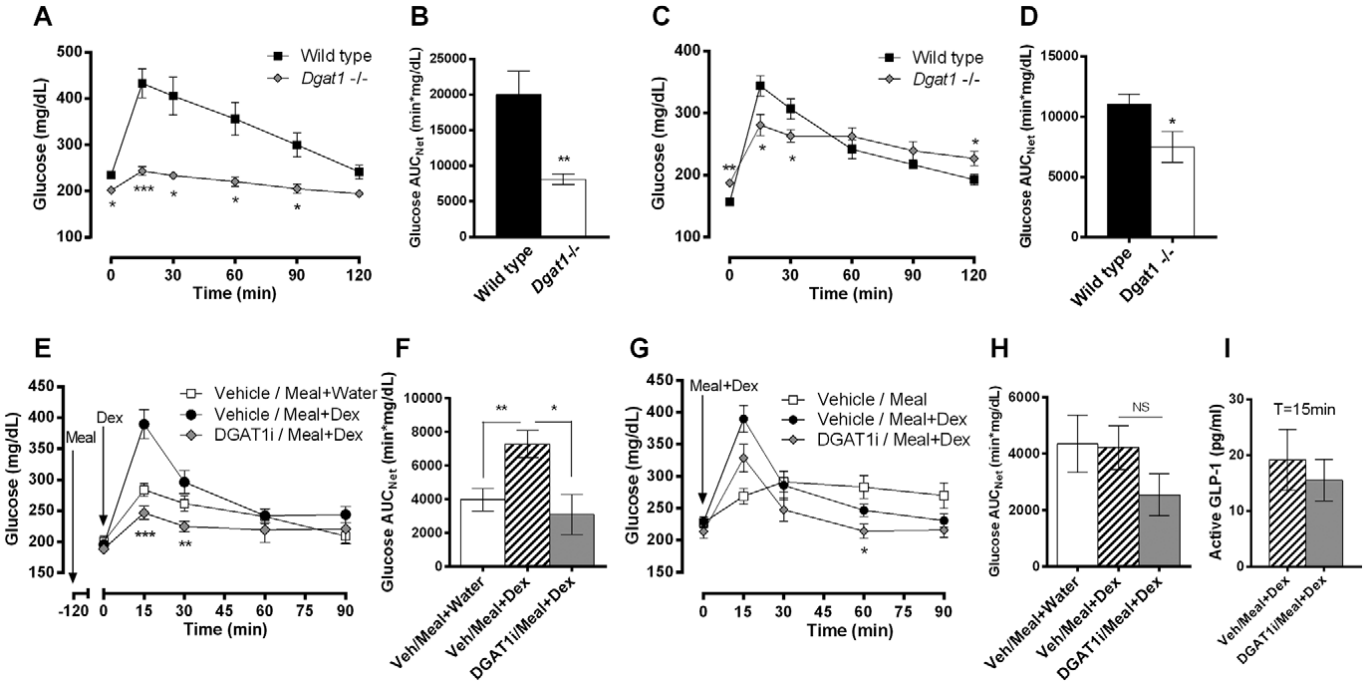
Body weight and food intake of gKD mice on HFD. Male gKD mice and wild type littermate raised no normal chow diet were switched to HFD at 4 months of age, and (A) body weight and (B) food intake were monitored for 8 weeks. No statistical significant difference was observed. N = 17–18.

gKD mice showed normal body weight and food intake on chow diet (data not shown) and on high-fat diet (HFD) (Figure 5A, B), consistent with the lack of effects on physiological gut hormone regulation. These data suggest that gut-selective inhibition DGAT1 by approximately 75% is insufficient for weight loss.

### Combined effects of DGAT1 with DPP-4 inhibition

**Figure 6:**
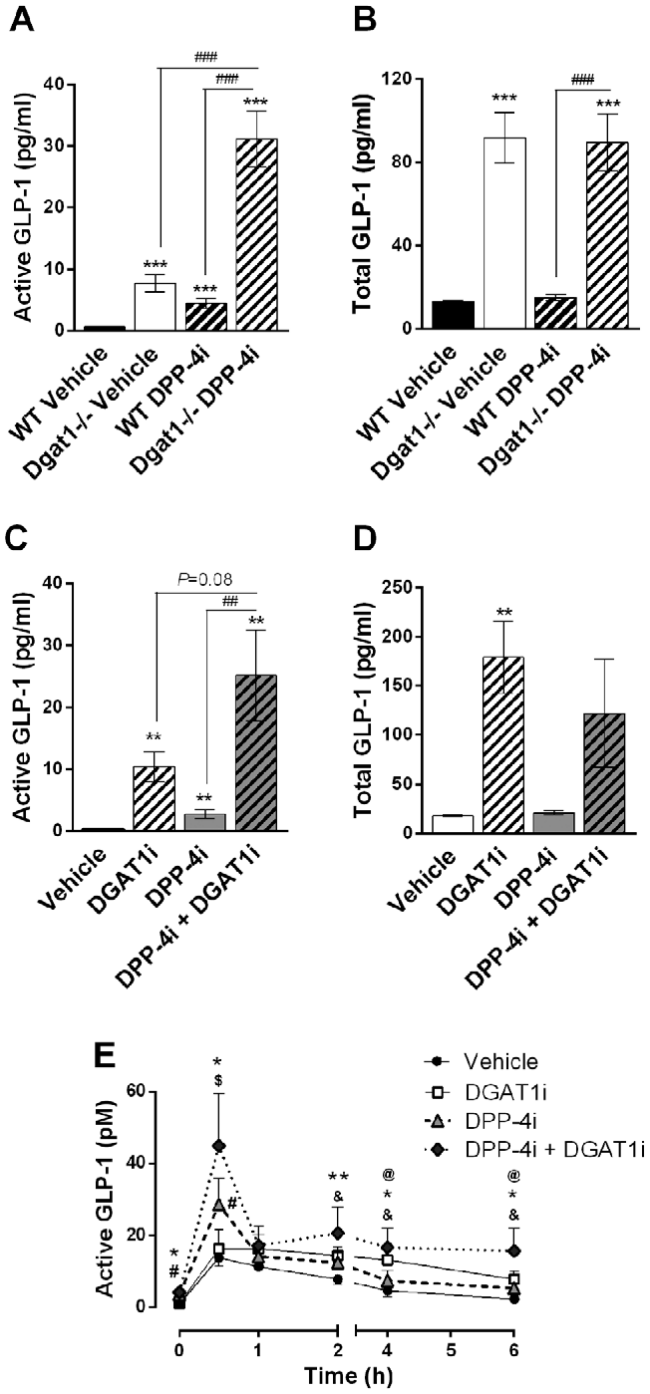
Combined effects of DGAT1 with DPP-4 inhibition on GLP-1. (A, B) 3-month-old lean *Dgat1*−/− and wild type mice were fasted overnight, then p.o. dosed with vehicle or a DPP-4 inhibitor (10 mg/kg) at -1 h. At time 0, mice were p.o. dosed with a meal containing 28.9% lipid load. (A) Plasma active GLP-1 and (B) total GLP-1 were measured at 2 h after meal challenge. N = 8. **P<0.01, ***P<0.001 vs. vehicle. ^###^P<0.001 between indicated groups. (C, D) 5-month-old DIO mice were p.o. dosed vehicle or DGATi (1 mg/kg) at -18 h and fasted overnight, then received vehicle or DPP-4 inhibitor (10 mg/kg) treatment at -1 h. At time 0, mice were p.o. dosed a meal with 28.9% lipid load, and plasma (C) active GLP-1 and (D) total GLP-1 were measured at 2h after meal challenge. N = 8. **P<0.01, ***P<0.001 vs. vehicle. ^##^P<0.01 between indicated groups. (E) Lean male beagle dogs were fasted overnight, then p.o. dosed the next morning (time -2 h) with vehicle, DGAT1i (0.3 mg/kg), DPP-4i (3 mg/kg), or a combination of DGAT1i and DPP-4i. At time 0, animals were p.o. dosed heavy cream (4 ml/kg). Serial blood samples were collected at indicated time points, and plasma active GLP-1 was measured. N = 4 for vehicle treatment, N = 6 for all other groups. *P<0.05, **P<0.01 DGAT1i/DPP-4i combo vs. vehicle. ^#^P<0.05 DPP-4i vs. vehicle. ^&^P<0.05 DGAT1i vs. vehicle. ^$^P<0.05 combo vs. DGAT1i alone. ^@^P<0.05 combo vs. DPP-4i alone.

Pharmacological inhibition of DPP-4 increases active GLP-1 by blocking its conversion into the inactive forms [20]. We therefore hypothesized that combined inhibition of DGAT1 and DPP-4 would result in an additive increase in active GLP-1. Indeed, at 2 h after meal challenge, *Dgat1*−/− mice treated with DPP-4i showed a 4-fold increase in active GLP-1 compared to vehicle-treated mice (Figure 6A). Importantly, total GLP-1 levels were not further increased by DPP-4i treatment (Figure 6B), as DPP-4i increases the stability of active GLP-1 rather than stimulates its secretion. Similarly, combination of DGAT1i and DPP-4i treatments in wild type mice led to further elevations in postprandial active GLP-1 compared to either compound alone (Figure 6C), while total GLP-1 levels were not increased by DPP-4i (Figure 6D). Combined treatment of DGAT1i and DPP-4i in lean beagle dogs also led to enhanced elevations in active GLP-1 compared to DPP-4i alone (Figure 6E), suggesting this combinatorial effect is conserved in higher species.

## Discussion

The key findings of this study are: (1) intestinal DGAT1 plays a major role in regulating plasma TG and gut hormones after a lipid-rich meal; (2) each of these parameters show different sensitivities to the degree of DGAT1 inhibition and meal lipid content, with TG excursion being the most sensitive–as it was attenuated in ∼50% DGAT1 deficiency (*Dgat1*+/− mice) with a lipid load as low as 19%, GLP-1 and PYY having intermediate sensitivities–as they were affected in 50–75% DGAT1 deficiency (*Dgat1*+/− and gKD mice) with high lipid loads, and GIP being the least sensitive–as it was only attenuated in >75% DGAT1 deficiency (gKD and *Dgat1*−/− mice) with high lipid loads present; and (3) combination of DGAT1 inhibition with DPP-4 inhibition results in further enhancements in active GLP-1.

Biochemical assay measuring DGAT enzymatic activity in wild type intestinal mucosa using TG synthesis as the readout showed a marked, albeit incomplete, inhibition by a DGAT1 inhibitor. This is consistent with reductions in postprandial TG excursion in *Dgat1*−/−, *Dgat1*+/−, and gKD mice, and provides conclusive evidence that DGAT1 activity in the intestine plays a key role in regulating TG resynthesis and secretion from enterocytes. Residual TG synthesis activity was observed in intestinal mucosa from *Dgat1*−/− mice. Taken together, these data suggest additional enzymes with DGAT-like activities contribute to TG synthesis in the small intestine in the absence of DGAT1. *Dgat1*+/− mice with a partial reduction in DGAT1 mRNA expression also showed a partial reduction in DGAT1i binding in intestinal mucosa, indicating that mRNA expression correlates well with DGAT1 enzyme activity. Furthermore, our findings are consistent with a recent report showing intestinal DGAT1 inhibition blocked postprandial TG excursion [18].

Examination of the gut hormone response to mixed meals containing varying amounts of lipid revealed an exquisitely sensitive interaction between meal content and the degree of DGAT1 inhibition. In the complete absence of DGAT1 activity (*Dgat1*−/− or DGAT1i treatment), meals with lipid load exceeding 19–29% were sufficient to trigger marked elevations in GLP-1 and PYY. On the other hand, 50–75% DGAT1 deficiency in the intestine (*Dgat1*+/− or gKD) did not affect GLP-1 or PYY after 19% lipid load, but led to elevations in GLP-1 and PYY when meal lipid content was higher. These data indicate that the residual intestinal DGAT1 activity in *Dgat1*+/− and gKD mice is relatively effective in processing dietary lipid substrates for TG synthesis. Conversely, when meal lipid content is above a certain threshold (53–100%), higher amounts of lipid substrates present in the intestine likely surpass the processing capacity of the residual DGAT1 enzyme. This could lead to carbon channeling into alternative pathways to form other lipid species (Liu et al., submitted), which in turn may act as signaling molecules to stimulate GLP-1 and PYY secretion. We consider meal challenges containing lipids exceeding 19.4% to be supraphysiological (Table 1). Thus, our data suggest that partial DGAT1 inhibition only has an impact on gut hormones in the presence of a supraphysiological oral lipid load, and near-complete DGAT1 inhibition is required to elevate postprandial GLP-1 and PYY under physiological conditions.

The threshold for attenuation of postprandial GIP appears distinct from that for GLP-1/PYY elevation. *Dgat1*+/− mice showed normal plasma GIP, even in the presence of 100% lipid, and gKD mice showed attenuated GIP induction following a meal containing 53% lipid load. Following a meal containing lower lipid content, postprandial GIP rapidly returned to baseline and was not further reduced by DGAT1 deficiency. The different requirement for DGAT1 function exhibited by GIP compared to GLP-1/PYY may reflect distinct proximodistal localization of enteroendocrine cells secreting these hormones. GIP-expressing cells are predominantly localized to the proximal small intestine. In contrast, GLP-1-expressing cells are less abundant in the proximal gut and more enriched in the distal small intestine and colon, while PYY-expressing cells are almost exclusively found in the colon [26]. Therefore, different enteroendocrine cell types may be exposed to different amounts and/or species of meal-derived lipid substrates and thus display different sensitivities to DGAT1 deficiency.

It's worth noting that gut-selective DGAT1 inhibition by ∼75% did not affect body weight and food intake. This finding is consistent with the observation that intestinal TG absorption and resynthesis are highly efficient [27], [28]. However, we cannot rule out the possibility that DGAT1 inhibition/loss-of-function in additional non-gut tissues is necessary to confer DIO resistance in *Dgat1*−/− or DGAT1i-treated mice. Future studies on tissue-specific DGAT1 knockout models will be required to address these possibilities.

Phase IIb clinical trial results for a DGAT1 inhibitor in obese patients with type 2 diabetes were recently reported, which showed modest reductions in HbA1c and body weight. Importantly, all efficacious doses were associated with dose-dependent increases in the incidences of diarrhea, nausea, and vomiting [29]. Consistent with these findings, a loss-of-function mutation in *DGAT1* was recently linked to congenital diarrheal disorder in a family of Ashkenazi Jewish descent [30]. Although the mechanisms underlying these gastrointestinal effects are not well understood, they are consistent with engagement of the GLP-1 pathway, which is likely also a major contributor to the efficacy on body weight and glycemia. Our findings that near-complete DGAT1 inhibition is required to elevate GLP-1 further indicate that it may not be feasible to achieve dose separation between efficacy and gastrointestinal adverse effects. Thus, DGAT1 inhibitor monotherapy is unlikely to have sufficient therapeutic margin to treat obesity and diabetes. On the other hand, DGAT1 inhibition likely has additional GLP-1-independent therapeutic benefits, such as improving dyslipidemia. Indeed, a recent study showed that intestinal DGAT1 inhibition blocked postprandial TG and retinyl ester excursion by inhibiting chylomicron secretion independent of delayed gastric emptying [18]. Thus, it's formally possible that certain metabolic benefits of DGAT1 inhibition could be dissociated from its GLP-1 secretagogue activity and gastrointestinal adverse effects.

Mechanisms that stimulate GLP-1 release are prime candidates for combination therapy with DPP-4 inhibition. Metformin increases GLP-1 synthesis and secretion, and leads to additive increases in active GLP-1 when combined with a DPP-4 inhibitor in rodents and man [31]. We evaluated DGAT1 inhibition in combination with a DPP-4 inhibitor in mice and dogs and showed further elevations in active GLP-1 in animals treated with combination therapy. Therefore, it's conceivable that the presence of a DPP-4 inhibitor may to improve the tolerability of DGAT1 inhibitors, potentially achieving the same increases in active GLP-1 with a lower degree of DGAT1 inhibition. Thus, the therapeutic potential of combination therapy with DPP-4 inhibition and DGAT1 inhibition warrants further investigation.

## Experimental Procedures

### Experimental animals

*Dgat1*−/− [16] males and *Dgat1*+/− females were used to produce *Dgat1*−/− and *Dgat1*+/− progeny on C57BL/6N background (Taconic Farms). Male *Dgat1*−/− and *Dgat1*+/− mice and age-matched C57BL/6N males were used for analyses. gKD mice were generated by pronuclear injection of a transgenic vector containing a 12.4 kb *Villin1* promoter [24] driving an EGFP-encoding sequence followed by a sequence encoding miR155-embedded *Dgat1* shRNA (Invitrogen) in C57BL/6N embryos. The resulting founder mice were confirmed for germline transmission and knockdown of DGAT1 mRNA expression in intestinal mucosa. Mice were genotyped using primers within the EGFP coding region: 5′-GACGGCGACGTAAACGGCCA-3′ and 5′-CGGTTCACCAGGGTGTCGCC-3′, which produce a 311 bp fragment in the presence of the transgene. gKD colony was maintained by transgenic to wild type breeding to generate gKD and non-transgenic littermate controls. Animals were maintained in a 12 h light/12 h dark cycle. Diets used were standard chow diet (Teklad 7012, Harlan Teklad) and high-fat diet (D12492, 60%kcal from fat, Research Diets). Body weight and food intake measurements were performed weekly in individually housed mice. All animal procedures were approved by the Merck Research Laboratories Institutional Animal Care and Use Committee (Rahway, NJ).

### Realtime PCR

Tissue RNA was extracted using reagents from Qiagen. Quantitative RT-PCR was performed using Taqman reagents (Applied Biosystems).

### DGAT1 enzymatic activity and inhibitor binding assays

Mouse intestinal mucosa was used as the DGAT1 source. Briefly, mucosa was homogenized in 200 mM Tris-HCl (pH7), 200 mM sucrose, 200 mM MgCl_2_ buffer containing protease inhibitors. Microsomes were prepared in ice-cold buffer containing 10 mM HEPES, 12 mM MgSO_4_, 1 mM EGTA. Enzymatic assay was conducted in the presence of 20 µM ^14^C-oleoyl CoA (American Radiolabeled Chemicals), 100 µM diolein, 5% ethanol, 100 mM Tris-HCl (pH 7), 100 mM sucrose, 100 mM MgCl_2_. Labeled TG was extracted by chloroform-methanol, separated by TLC in hexane-diethylether-acetic acid, and quantified with a Typhoon 9410 Variable Mode imager. To determine saturable and specific binding of ^3^H-labeled inhibitor to DGAT1, microsomes were incubated in the absence or presence of ligands in 100 mM Tris (pH 7.4), 100 mM sucrose, 100 mM MgCl_2_. For competition binding, final concentrations of ^3^H-labeled inhibitor were 0.3 nM and competing cold inhibitor was added in the range of 0.02–1000 nM, with final DMSO concentration ≤1%. Bound inhibitor was captured on 25 mM GF/C filter coated with 0.5% polyethyleneamine, washed, and quantified in Ecolume (MPBio) with a TriCarb 2900TR liquid scintillation analyzer (Perkin Elmer). Bmax and Kd were determined with Graphpad Prism using one-site specific binding classical equation for nonlinear regression analysis. Competition data were analyzed with using sigmoidal dose-response (variable slope) classical equation for nonlinear regression analysis. All chemicals were obtained from Sigma-Aldrich unless otherwise specified.

### Meal challenge in mice

Mice were fasted overnight but allowed free access to water. At time 0, mice were dosed *per os* (p.o.) with corn oil (Fisher Scientific) or an admixture of corn oil and Ensure-plus (1.48 kcal/ml, 28.5%kcal from fat, Abbot) at 10 ml/kg ad libitum body weight. For TG measurements, blood was collected via unrestrained tail bleeds into heparinized microcapillaries. For hormone measurements, blood was collected via cardiac or submandibular bleeds into EDTA-tubes containing DPP-4 inhibitor (Millipore) and protease inhibitor cocktail.

### Meal challenge in dogs

2 to 6-year-old lean male beagle dogs were fasted overnight then treated with indicated compounds the next morning. At time 0, animals were p.o. dosed heavy cream (4 ml/kg). Serial blood samples were collected at indicated time points into EDTA-tubes containing DPP-4 inhibitor and protease inhibitor cocktail.

### Plasma measurements

Plasma TG was measured using an Infinity Triglyceride kit (Thermo). Total GLP-1 and active GLP-1 were measured using immunoassays (Meso Scale Discovery). Total GIP, active PYY, and insulin were measured using the Milliplex gut hormone panel (Millipore).

### Statistical analyses

Data were analyzed by Student's t test or one-way ANOVA with Bonferroni post test. P values less than 0.05 were considered significant.
